# Attachment and Autism Spectrum Disorder (Without Intellectual Disability) During Middle Childhood: In Search of the Missing Piece

**DOI:** 10.3389/fpsyg.2021.662024

**Published:** 2021-05-28

**Authors:** Michele Giannotti, Simona de Falco

**Affiliations:** Department of Psychology and Cognitive Sciences, University of Trento, Rovereto, Italy

**Keywords:** Autism Spectrum Disorder, attachment representations, internal working models, school-age, middle childhood

## Introduction

The study of Autism Spectrum Disorder (ASD) represents one of the most challenging areas for attachment research in view of the complex interrelation between socio-communication impairments and attachment processes (McKenzie and Dallos, 2017; Vivanti and Nuske, 2017). The fascinating debate, which focuses on whether ASD hampers the development of secure attachment, has not received sufficient attention so far. Prior studies often focused on attachment behaviors in preschoolers with ASD, whereas the study of attachment representations in older children remains mainly unexplored. The most recent systematic review (Teague et al., 2017) highlighted crucial unresolved questions in the study of attachment and ASD with reference to the paucity of data about predictors, correlates and outcomes of attachment in children with ASD. For instance, there is mixed evidence on the association between parental sensitivity and child attachment security, which has been widely documented in typically developing children (Van IJzendoorn et al., 2007). Importantly, the need to adopt a longitudinal perspective to examine the association between attachment and emotional or behavioral difficulties has been also highlighted. To this aim, the study of attachment requires a developmental lens based on age-appropriate methods. In this regard, research has not yet addressed the importance of investigating attachment representations in middle childhood and adolescence. In the last decade, only a limited number of studies on attachment and ASD has been published on children without intellectual disabilities during middle childhood (Bauminger et al., 2010; Chandler and Dissanayake, 2014; Keenan et al., 2016; Sivaratnam et al., 2018). The assessment of attachment in each study is based on questionnaires such as the Inventory of Parent and Peer Attachment (IPPA, Armsden and Greenberg, 1987) and/or the Security Scale (Kerns et al., 1996). Age range and number of the participants vary across studies, including only children from 6 to 14 years with High-functioning ASD. ASD sample size across studies was fairly small, ranging from 21 children with ASD in the Chandler and Dissanayake’s study to 44 children of Bauminger et al. (2010), which, however, includes participants from two countries (24 from Israel and 20 from the United States).

These studies revealed no group differences on security of attachment to their caregivers between children with ASD and children typical development (TD), confirming previous findings on early childhood (Teague et al., 2017). Nonetheless, some relevant issues concerning the quality of attachment in school-aged children with ASD are still underinvestigated. Firstly, in contrast to the subsequent studies, Bauminger et al. (2010) found significant differences between groups (ASD vs. TD) using the IPPA, which is considered the most valid and reliable attachment self-reported measure during middle childhood (Jewell et al., 2019). This contradictory result may be due to the fact that a larger sample of size has been considered (*n* = 44) compared to the subsequent studies.

Thus, despite evidence on the lack of group differences are accumulating, this result needs to be further confirmed using larger samples. In addition, self-reported measures have been criticized for not sufficiently capturing the complexity of this phenomenon, with particular reference to the level of attachment implicit representations. Moreover, previous findings (Chandler and Dissanayake, 2014) only revealed a “negligible” parent-child agreement on the IPPA scores, suggesting that the use of defensive strategies as well as diminished self-awareness in these children could raise further doubts about the validity of attachment questionnaires in this specific population.

More importantly, prior research, except for case studies (Brewerton et al., 2017), exclusively used questionnaires, which are only based on child perception of the quality of parent-child relationship and thereby on conscious knowledge (evaluative-declarative). By contrast, implicit internal representations are mainly automated and unconscious, reflecting the transformation of a dyadic quality into an individual characteristic. The internalization of dyadic experiences into a representational system occurs during childhood, becoming more elaborated as language ability increases across development (Bowlby, 1973; Bretherton and Munholland, 2008). This transition led to the construction of a cognitive form of attachment (Kraemer, 1992) originally defined as internal working models (IWM; Bowlby, 1973). In this regard, interpersonal expectations, self-beliefs, perspective-taking and interpersonal schemas are strictly connected to information surrounding the relation between the self and the context (Thompson, 2008; Crittenden, 2016).

Following a dual process framework, observer-rated narratives assess non-conscious implicit processes, whereas self-reported questionnaires are based on child conscious explicit representations. The explicit conscious processes have been identified as externally-oriented high-order cognitions which follow the development of the implicit internally-oriented core processes related to one’s self-concept and their relationship with others (Howe and Courage, 1997). Prior research on adults highlighted that these distinct processes also correlated with different brain activation (Yaseen et al., 2016). Respectively, cortical activity underpins the explicit conscious representations of the attachment figure, whereas narratives methods based on implicit models of attachment are associated with subcortical structures (Northoff and Panksepp, 2008). Focusing on middle childhood, attachment narratives and self-reported measures were found to be complementary in predicting psychological symptoms in school-age children (Borelli et al., 2016). In fact, the variety of methods used for the assessment of attachment during middle childhood may reflect different conceptualization and can be used concurrently with the aim to evaluate different components of the common construct and neural network (Bosmans and Kerns, 2015; Yaseen et al., 2016). Although different methods (e.g., semi-projective, autobiographical interview, story stems, family drawing) are available for the assessment of implicit attachment representations in children and adolescents, to the best of our knowledge, there are no quantitative studies on middle childhood that filled this critical gap in ASD literature. Therefore, in this opinion article, we discuss the considerable prospects associated with the study of implicit attachment representations in children with ASD, highlighting the importance to cover this unaddressed topic. Given that attachment narrative-based assessment in this developmental stage often requires verbal abilities, we focus on children with ASD without intellectual disabilities.

## Attachment Representations in ASD: What We Still Need to Know

Despite the lack of data, it has been suggested that individuals with a diagnosis of ASD may experience the construction of internal models of attachment as challenging (Rogers et al., 1993; Chandler and Dissanayake, 2014). Difficulties in interpersonal relatedness and intersubjectivity (Stern, 2004), which characterize ASD social-communication impairment could make it more difficult for both children and parents to give meaning and predict each other’s behaviors, therefore complicating the development of balanced implicit representations. These mechanisms may also undermine sensitive and responsive caregiving increasing the frequency of relational impasses, including non-consequential, non-attuned and unrewarding interactions (Guo et al., 2017). In fact, since the link between parental sensitivity and attachment security has not been confirmed in ASD literature, in this case the transmission of attachment representations may be disrupted by difficulties in mind-minded, insightful and reflective caregiving (Teague et al., 2017).

Following this hypothesis, although children with ASD (without intellectual disability) do not differ significantly from controls in attachment security assessed through observational procedure, it is plausible to expect a discontinuity from attachment behaviors to the level of representations. Moreover, additional factors may hinder the organization of adaptive and reflective mental processing with respect to attachment representations and interpersonal schemas. Specifically, child unusual brain structure and functional connectivity (Crittenden, 2017; Cole et al., 2019), atypical neuroendocrine processes and dysregulation (Naber et al., 2007; Sivaratnam et al., 2015) hypo or hyper-sensitivity to sensory stimulation (Marco et al., 2011) difficulties in multisensory integration (Stevenson et al., 2014) can pose multiple challenges for children with ASD. Moreover, an additional source of risk is the frequent exposure to potential traumatic events. Berg et al. (2016), documented a significant association between ASD and the occurrence of Adverse Childhood Experiences (ACEs), showing higher rates of income insufficiency, parental divorce/separation, exposure to neighborhood violence and household mental illness in individuals with ASD compared to controls. Research has also highlighted that the experience of bullying is 3–4 times more frequent for children with ASD compared to their typically developing peers (Hoover and Kaufman, 2018). Taken together, these factors may interfere with the development of a resolved attachment status of children with ASD, given that past dangerous events may activate unresolved traumatic memories affecting individuals’ strategic current functioning (Crittenden, 2016).

Interestingly, to date, only one study (Taylor et al., 2008) investigated attachment implicit representations in ASD, focusing on adults without intellectual disability. The Adult Attachment Interview (AAI, George Kaplan and Main) was used to assess pattern of attachment of 20 adults with High-functioning Autism and 20 matched controls. Results showed that only a 15% of the ASD sample has been classified as secure, which is a lower rate compared to those found in early childhood (Rutgers et al., 2004; Teague et al., 2017). AAI transcripts of ASD participants were less coherent highlighting lower levels of reflective functioning compared to matched controls. The rate of attachment security in this study is similar to those of other clinical samples as highlighted by previous metanalytic findings (van IJzendoorn and Bakermans-Kranenburg, 1996). Additionally, these findings confirmed that individuals with ASD can develop secure attachment representations, albeit with a lesser extent compared to the control group. Beyond this pioneering study, these hypotheses have not been tested in ASD literature, thus further investigations need to be conducted to replicate this result in childhood and adolescence.

Furthermore, it is of particular interest to assess attachment outcomes through different methods that are not directly influenced by individual strategic control or introspective difficulties associated with ASD. The use of implicit measures may provide useful and reliable data on children’s representations of attachment figures as well as on current state of mind with respect to attachment in ASD. Notably, internal models of self and others shape information processing as well as emotion and behavior regulation, influencing the way in which children process and respond to social stimuli (Zimmermann and Iwanski, 2015). Biased expectations of others’ intentions related to at-risk information processing may increase emotional, social difficulties and the risk of psychiatric comorbidity, that constitute critical aspects for the development of children with ASD. Thus, reaching a more comprehensive knowledge about implicit attachment representations during middle childhood may help to better understand ASD attachment difficulties. This could clarify children’s needs of protection and comfort before the sensitive period of transition to adolescence, offering valuable information to personalized psychological interventions, educational support and to foster attachment relationships with parents and peers.

Moreover, the association between implicit representations, behavioral and emotional problems could shed light on the protective mechanisms related to attachment organization in children with ASD. In this regard, there is growing interest in identifying potential predictors of positive long-term developmental trajectories, adaptive functioning and health-related quality of life in individuals with ASD (Pugliese et al., 2016; Adams et al., 2019). Information on quality of attachment representations may provide a significant contribution to foster mental health, reducing the risk of co-occurring psychiatric disorders and unresolved traumatic experiences in individuals with ASD. Studies on school-age children with typical development, showed the significant association between insecure attachment representations and child externalizing-internalizing symptoms (Moss et al., 2009). Although there are no studies that support this association in children with ASD, a recent longitudinal study (Rozga et al., 2018) suggests that the sequelae of attachment security in ASD may be similar to those documented for TD children. Thus, enhancing an internal sense of security, resilience and adaptation in children with ASD should represent a priority of autism agenda, for which the understanding of attachment representations constitutes a necessary precondition.

In conclusion, our recommendation is to complement the limited body of research on attachment and ASD during middle childhood by including the assessment of implicit representations, their predictors, correlates and outcomes. Future research on children with ASD in middle childhood should consider a more global assessment of attachment combining and comparing different sources of information (e.g., parent-reported, self-reported, observer-rated). This challenge represents an intriguing opportunity to renew the scientific debate on attachment and ASD, which cannot be regarded as comprehensive, bearing in mind the lack of data and the multiple unanswered questions on implicit representations.

## Author Contributions

MG contributed to the conceptualization of the article and drafted the manuscript. SdF substantially contributed to the conception of the manuscript and revised it critically for important intellectual content. Both authors contributed to the article and approved the submitted version.

## Conflict of Interest

The authors declare that the research was conducted in the absence of any commercial or financial relationships that could be construed as a potential conflict of interest.
